# Foxp3 Post-translational Modifications and Treg Suppressive Activity

**DOI:** 10.3389/fimmu.2019.02486

**Published:** 2019-10-18

**Authors:** Guoping Deng, Xiaomin Song, Shigeyoshi Fujimoto, Ciriaco A. Piccirillo, Yasuhiro Nagai, Mark I. Greene

**Affiliations:** ^1^Department of Immunology, Peking University Health Science Center, Beijing, China; ^2^State Key Laboratory of Molecular Biology, CAS Center for Excellence in Molecular Cell Science, Institute of Biochemistry and Cell Biology, Chinese Academy of Sciences, Shanghai, China; ^3^Seishin Medical Group, Takara Clinic, Tokyo, Japan; ^4^Department of Microbiology and Immunology, McGill University, Montréal, QC, Canada; ^5^Centre of Excellence in Translational Immunology (CETI), Research Institute of the McGill University Health Centre, Montréal, QC, Canada; ^6^Department of Pathology and Laboratory Medicine, Perelman School of Medicine, University of Pennsylvania, Philadelphia, PA, United States

**Keywords:** regulatory T cells, Foxp3, post-translational modification, phosphorylation, O-GlcNAcylation, acetylation, ubiquitylation, methylation

## Abstract

Regulatory T cells (Tregs) are engaged in maintaining immune homeostasis and preventing autoimmunity. Treg cells include thymic Treg cells and peripheral Treg cells, both of which can suppress the immune response via multiple distinct mechanisms. The differentiation, proliferation, suppressive function and survival of Treg cells are affected by distinct energy metabolic programs. Tissue-resident Treg cells hold unique features in comparison with the lymphoid organ Treg cells. Foxp3 transcription factor is a lineage master regulator for Treg cell development and suppressive activity. Accumulating evidence indicates that the activity of Foxp3 protein is modulated by various post-translational modifications (PTMs), including phosphorylation, O-GlcNAcylation, acetylation, ubiquitylation and methylation. These modifications affect multiple aspects of Foxp3 function. In this review, we define features of Treg cells and roles of Foxp3 in Treg biology, and summarize current research in PTMs of Foxp3 protein involved in modulating Treg function. This review also attempts to define Foxp3 dimer modifications relevant to mediating Foxp3 activity and Treg suppression. Understanding Foxp3 protein features and modulation mechanisms may help in the design of rational therapies for immune diseases and cancer.

## Phenotypical Features of Regulatory T Cells

### Origins, Lineage Definition and Classification, Suppression Mechanism, Epigenetic and Metabolic Features of Treg Cells

CD4^+^CD25^+^ T cells have been identified as a suppressive T cell subset, which have a dominant role in mediating peripheral immune tolerance (1). The Treg population includes two main subsets according to their origins: thymic Treg cells (tTregs) develop with stimulations of self-antigens, cytokines, and costimulatory molecules in thymus; peripheral Treg cells (pTregs) are differentiated from naïve T cells by the act of cytokine TGF-β in periphery. Both tTregs and pTregs require expression of the Foxp3 transcription factor to maintain their lineage stability and effective suppression of immune responses (2, 3).

The definition of a stable Treg lineage can be evaluated by several features, including constitutive expression of Foxp3 and a variety of Treg feature markers (4). Expression of Foxp3 is a prerequisite for Treg lineage commitment and maintenance. Foxp3 expression relies on demethylation of the conserved non-coding sequence 2 (CNS2, also known as the Treg cell-specific demethylated region TSDR) in the *Foxp3* locus. A deletion of CNS2 results in loss of Foxp3 expression during Treg cell expansion and destabilizes Treg cells (5–7). High-resolution quantitative proteomics and transcriptomics approaches have revealed that expression patterns of the core Treg properties, including CD25, CTLA-4, Helios, and *FOXP3* gene TSDR methylation, appear relatively stable in culture *in vitro* (8). The role of Foxp3 in Treg function will be discussed below. Moreover, Treg cells are endowed with unique processes to rapidly respond to environmental cues, and can achieve this through distinct mechanisms of regulation of global or gene-specific mRNA translation. Unlike gene transcription, translational regulation is advantageous for environmental-sensing as it provides a rapid and energetically favorable mechanism to shape the proteome of a given cell, and to tailer cell function to the extracellular context (9). Indeed, distinct translational signatures distinguish Treg and Teff cells (10).

Treg cells are phenotypically diverse in migration, homeostasis, and function (11). Tregs are divided into CD44^low^CD62L^high^ central Tregs (cTregs) and CD44^high^CD62L^low^ effector Tregs (eTregs). cTregs are quiescent, IL-2 signaling dependent and long-lived, and they function in the secondary lymphoid tissues to suppress T cell priming; in contrast, eTregs are highly activated and ICOS signaling dependent with potent suppressive function in specific non-lymphoid tissues to dampen immune responses (12). eTregs have increased mTORC1 signaling and glycolysis compared with cTregs. Consistently, inhibition of mTORC1 activity by administration of rapamycin (mTORC1 inhibitor) promotes generation of long-lived cTreg cells *in vivo* (13). Treg cells lacking Ndfip1, a coactivator of Nedd4-family E3 ubiquitin ligases, elevate mTORC1 signaling and glycolysis, which increases eTreg cells but impairs Treg stability in terms of Foxp3 expression and pro-inflammatory cytokine production (14).

Treg cells suppress immune response via multiple mechanisms [as reviewed in (15–17)]. Treg cells highly express CD25 (the IL-2 receptor α-chain, IL-2Rα) and may compete with effector T cells leading to consumption of cytokine IL-2 (18). Treatment with low-dose rhIL-2 selectively promotes Treg frequency and function, and ameliorates diseases in patients with systemic lupus erythematosus (SLE) (19). The constitutive expression of CD25, a direct target of Foxp3, is essential to engage a strong STAT5 signal for Treg proliferation, survival, and Foxp3 expression (20). CTLA-4 activation can down-regulate CD80 and CD86 expression on antigen-presenting cells (21). Treg cells also produce inhibitory cytokines, IL-10, TGF-β, and IL-35, to enhance immune tolerance along with cell-contact suppression (22–24). Treg cells may mediate specific suppression by depleting cognate peptide-MHC class II from dendritic cells *in vivo* (25). Of note, Treg cells recognize cognate antigen and require T cell receptor (TCR) signaling for optimal activation, differentiation, and function (26). Polyclonal expanded Treg cell mixed populations exhibit suppressive potency for certain autoimmune diseases (27). Engineering Treg cells with antigen-specific TCR appears to lead to antigen-specific suppression with increased potency (28).

Treg cells exploit distinct energy metabolism programs for their differentiation, proliferation, suppressive function, and survival (29, 30). Rather than glucose metabolism, Treg cells have activated AMP-activated protein kinase (AMPK) and use lipid oxidation as an energy source. AMPK stimulation by Met can decrease Glut1 and increase Treg generation (31). Further proteomic analysis showed that fresh-isolated human Treg cells are highly glycolytic, while non-proliferating Tconv cells mainly use fatty-acid oxidation (FAO) as an energy source. When cultured *in vitro*, Treg proliferation and suppression require both glycolysis and FAO, while Tconv cells mainly rely on glucose metabolism for proliferation and function. This finding indicates that Treg cells and Tconv cells may adopt different metabolic programs *in vitro* and *in vivo* (32). Treg cells cannot only use anabolic glycolysis to produce sufficient fundamental building blocks to fuel cell expansion, but also efficiently generate ATP energy via catabolic fatty acid oxidation (FAO) driven oxidative phosphorylation (OXPHOS) by the mitochondria to support activation and suppression function (33).

Treg cells have greater mitochondrial mass and higher ROS production than Tconv cells. Tregs are more vulnerable to OXPHOS inhibition, which underscores the unique metabolic features of Treg cell (34). Loss of subunit of the mitochondrial complex III RISP in Treg cells diminishes oxygen consumption rate (OCR), but increases in glycolytic flux. Treg cells require the activity of mitochondrial complex III to maintain Treg feature gene expression and suppression (35). Foxp3 expression increases mitochondrial respiratory capacity and promotes ATP generation through oxidative phosphorylation. Increased fatty acid metabolism can protect Treg cells from fatty acid–induced apoptosis accordingly (36). Toll-like receptor (TLR) signals activate PI(3)K-Akt-mTORC1 signaling and glycolysis to promote cell proliferation, however, Foxp3 opposes PI(3)K-Akt-mTORC1 signaling but increases oxidative phosphorylation for effector function. Thus, Foxp3 and inflammatory TLR signals control the proliferation and suppressive function of Treg cells through balancing the metabolic activity between glycolysis and oxidative phosphorylation (37). Genetic deletion of mTOR gene (*Frap1*) or pharmacological inhibition of mTOR activity by rapamycin promotes Treg cell maintenance (38, 39). A recent study shows that the deficiency of Foxp3 dysregulates mTORC2 signaling, which promotes aerobic glycolysis and oxidative phosphorylation. Genetic deletion of the mTORC2 adaptor gene *Rictor* or pharmacological treatment with mTOR inhibitors antagonizes the Teff cell-like program and restores the suppressive function of *Foxp3*-deficient Treg cells (40).

### Features of Tissue-Resident Treg Cells

Treg cells adopt distinct patterns of tissue- or immune-context-specific suppression mechanisms (41). Treg function *in vivo* requires a timely recruitment and/or accumulation in non-lymphoid tissues where immune responses are frequently occurring. In these sites, Treg cells adapt their function (i.e., effector mechanism) based on local immune mediators (42). For instance, TNFα-induced dephosphorylation of FOXP3 at Ser-418 by protein phosphatase 1 (PP1) compromises Treg suppressive activity within the inflamed synovium, which is responsible for the pathogenicity of rheumatoid arthritis (43). Here, we attempt to review some unique features and underlying mechanisms of tissue-resident Treg cells in skin, visceral adipose tissue and tumor, which has attracted extensive research interests recently (44–46).

Skin is an immunologically active organ that protects against pathogen invasion but prevents collateral tissue damage (47). Both murine and human skin contain a large number of Tregs, which accumulate through multiple chemokines and ligands, such as CCR4, CCR6, CCL17, CCL20, and CCL22 (48, 49). Functionally, Tregs inhibit proinflammatory cytokine IFN-γ production and macrophage accumulation in wounded skin to facilitate cutaneous wound closure and healing (50). Skin-resident Treg cells localize to hair follicles and facilitate stem cell-mediated hair follicle regeneration through Notch-Jagged signaling pathway (51). Tregs contribute to the establishment of immune tolerance to commensal microbes in neonatal skin and colon (51–53).

Visceral adipose tissue (VAT)-resident Treg cells guard against abnormal inflammation of the adipose tissue in obesity, type 2 diabetes and atherosclerosis (54, 55). Progressively decreased frequency of Tregs in visceral adipose tissue leads to the adipose inflammation and insulin resistance in obese animal models, while the induction of Tregs by administration of IL-2 contained complex or anti-CD3 monoclonal antibody can ameliorate glucose tolerance and insulin sensitivity (56, 57). VAT-Treg cells show unique gene signatures implicated in leukocyte migration, extravasation, and cytokine production (56). Peroxisome-proliferator-activated receptor γ (PPARγ) acts as a crucial orchestrator for the accumulation, phenotype, and function of VAT-Treg cells; a deficiency of PPARγ in Treg cells down-regulates the specific VAT Treg gene expression signature and Treg frequency in the visceral adipose tissue particularly (58). VAT-Treg cells maintain adipose tissue homeostasis by catabolizing prostaglandin E2 (PGE2) into the metabolite 15-keto PGE2, which limits conventional T cell activation and proliferation in visceral adipose tissue (59).

Treg cells are often enriched in tumor tissue, and a high ratio of tumor-infiltrating Treg cells (TIL-Tregs) to effector T cells generally predicts poor clinical outcomes of certain types of cancers, including ovarian cancer, breast cancer, melanoma, and hepatocellular carcinoma (60). Depleting intra-tumoral Tregs by treatment with immune checkpoint inhibitor monoclonal antibody against cytotoxic T lymphocyte–associated antigen 4 (anti-CTLA-4) promotes anti-tumor immune activity (61, 62). A recent study shows that the antitumor efficacy of anti-CTLA-4 can be enhanced by the combination therapy with Toll-like receptor 1/2 (TLR1/2) ligand via a FcγRIV-dependent manner (63). The origins of TIL-Tregs are diverse, including: (1) selective recruitment to tumor sites; (2) conversion from conventional CD4^+^ T cells; (3) Treg cell expansion (64). TIL-Tregs exhibit Foxp3 TSDR hypomethylation, and are highly proliferated and apoptotic due to the oxidative stress within tumor lesions (65, 66). Treg cells can thrive in tissues with ischemic injury or the tumor microenvironment, characterized by low-glucose and high-lactate. Treg cells favor oxidation of L-lactate to pyruvate to increase the intracellular NAD:NADH ratio, and resist the suppressive effects of L-lactate on cell proliferation (67). In addition, Tregs are endowed with the relative advantage of a circuitry of glycolysis, fatty acid synthesis, and oxidation to prevail over conventional T cells in the hostile tumor microenvironment (68).

Overall, tissue-resident Treg cells play unique roles in distinct non-lymphoid tissues via multiple molecular mechanisms, including regulation by post-translational modification of FOXP3 protein (which will be discussed in detail in the section of “Post-translational modifications of Foxp3”). It holds promise to harness the behavior and function of tissue-resident Tregs, specifically to treat certain immune disorders, such as skin autoimmunity and regeneration, obesity, type 2 diabetes and cancer.

## FOXP3 Is a Dominant Regulator of Treg Cells

The Forkhead box (FOX) protein superfamily of transcriptional regulators play pleiotropic roles in cell proliferation, differentiation, survival, and apoptosis during embryonic development and homeostasis of adult tissues (69). The FOXP family include FOXP1, FOXP2, FOXP3, and FOXP4 members. Foxp1 interacts with and regulates Foxp3 chromatin binding to coordinate Treg cell suppressive function and homeostasis (70–72). In humans, the FOXP3 gene (originally named JM2 or Scurfin) is found on the X-chromosome at Xp11.23-Xq13.3 (73). Genetic analysis has demonstrated that Treg cell dysfunction caused by the FOXP3 mutation is responsible for the immune dysregulation polyendocrinopathy enteropathy X-linked syndrome [IPEX, also called X-linked autoimmunity–allergic dysregulation syndrome (XLAAD)], a recessive immune disorder occurring in newborns and children (74). In mice, conditional deletion of Foxp3 in CD4^+^ T cells leads to fatally lymphoproliferative autoimmunity, which includes high IgE levels, enteropathy, type 1 diabetes and failure to thrive, while ectopic expression of Foxp3 can re-program conventional CD4^+^ T cells as anti-inflammatory Treg cells (75).

The Foxp3 protein contains multiple structural domains. There is a proline-rich N-terminal domain that interacts with many molecular factors to regulate transcriptional activity. Another central zinc-finger and leucine-zipper domain is involved in oligomer formation, and a conserved C-terminal forkhead/winged helix domain (FKH) is responsible for DNA binding (76). Chromatin immuno-precipitation combining genome-wide analysis revealed that Foxp3 interacts with up to ~700 genes. Interestingly, Foxp3 can activate or repress its target genes to cooperatively regulate Treg cell development, function, and homeostasis (77, 78). Foxp3 protein complexes define the transcriptional network of Treg cells (79). Foxp3 forms heterogeneous super-complexes of 400–800 kDa and associates with 361 partner proteins of which ~30% are transcription related. These proteins include Foxp1, Foxp4, Stat3, IKZF1, Runx1, and GATA-3 (71, 72). These interactions highlight the master role of Foxp3 in programming Treg cell lineage.

FOXP3 mutations that occur in IPEX patients include missense point mutations, frameshift, and missplicing, causing a premature stop codon, as well as mutations in the polyadenylation site. The missense mutations can preserve a normal or reduced expression of FOXP3 in IPEX patients. Although various distinct FOXP3 mutations have been reported with IPEX patients, individuals with the same mutation can develop disease manifestations that are not similar, indicating heterogeneity of severity among IPEX cases (80). IPEX patients may present with different intestinal lesions (81), and similar genotypes come out with different symptoms and severity (82); which suggests the complex relevance of genotype and phenotype in IPEX patients, and reflects complex intracellular interactions and post-translational modifications of FOXP3 (83).

## Post-translational Modifications of Foxp3

**Figure 1:**
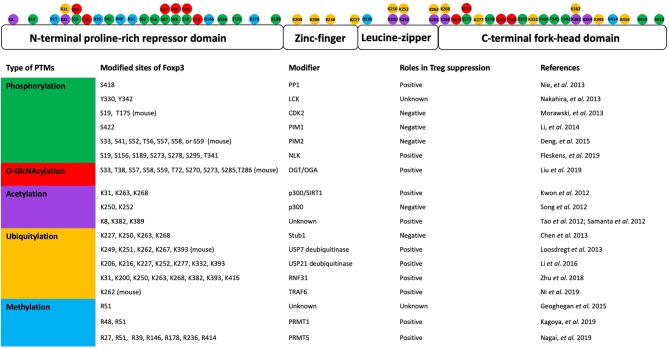
Post-translational modifications of Foxp3 and their roles in Treg suppression. A schematic representation shows the Foxp3 protein structure domains and post-translational modification sites **(top)**. The table lists the kind of modification, modified sites, modifier enzymes, and roles in Treg suppression **(bottom)** (Guoping Deng and Mark I. Greene).

Post-translational modifications (PTMs) of proteins link cellular signals to the functional properties. The transcriptional activity of Foxp3 can be modulated by various post-translational modifications, such as phosphorylation, O-GlcNAcylation, acetylation, ubiquitination, and methylation (Figure 1).

### Phosphorylation and Dephosphorylation of Foxp3

Protein phosphorylation, occurring at serine, threonine or tyrosine resides of proteins, is a reversible and transient modification catalyzed by certain kinases and phosphatases. Protein phosphorylation is involved in protein intracellular stability, interaction, and localization. TCR engagement triggers a signaling cascade with rapid phosphorylation events, and reprograms the proteomes and bioenergetic features for T cell activation, proliferation, and differentiation (84). In humans, TCR stimulation can induce transient FOXP3 expression (85). TCR signaling enhances Foxp3 phosphorylation through incubation of anti-CD3/CD28 antibodies or pharmacological treatment with PMA (phorbol 12-myristate 13-acetate) and Ionomycin (86). A recent report found that TCR stimulation can activate TAK1-NLK signaling, leading to phosphorylation of Foxp3, which decreases its interaction with the STUB1 E3-ubiquitin ligase to modify ubiquitination and proteasome-mediated degradation rates. Using mass spectrometry, seven distinct phosphorylated residues (S19, S156, S189, S273, S278, S295, and T341) of Foxp3 have been identified upon the co-expression of NLK in cell line. Although there may be other substrates targeted by NLK kinase other than Foxp3, Treg cell-specific NLK deficiency results in auto-inflammation and renders animals susceptible to induced experimental autoimmune encephalomyelitis (EAE) (87).

Levels of the inhibitor of the cyclin-dependent kinases (CDKs) p27kip1 are up-regulated in anergic T cells. CDK2 is a direct target of p27kip1 and CDK2 promotes cytokine production by CD4^+^ T cells, while limiting Treg function. Treg cells lacking CDK2 had increased capacity to inhibit Tconv cell proliferation and ameliorate immunopathogenesis in colitis (88). The N-terminal domain of Foxp3 protein contains several putative CDK motifs. CDK2 kinase phosphorylates Ser-19 and Thr-175 residues of Foxp3. Mutation of CDK motifs via a substitution of Ser/Thr to Ala increases Foxp3 protein stability and transcriptional activity, which manifests that Foxp3 phosphorylation by CDK2 kinase negatively regulates Treg function (89). These findings demonstrate that CDK2 negatively regulates Treg function and peripheral tolerance by phosphorylating Foxp3.

The Foxp3 N-terminal domain is important for Foxp3 transcriptional repression and nuclear transport (90, 91). Pim-2 kinase, an oncogenic serine/threonine kinase, can phosphorylate multiple sites of Foxp3 N-terminal domain as identified by mass spectrum analyses. The deficiency of Pim-2 increases Treg suppression in *in vitro* assay, and *Pim-2*-deficient mice appear resistant to DSS-induced acute colitis (92). Pim-2 kinase can promote rapamycin-resistant survival, growth and proliferation of lymphocytes, including Treg cells (93, 94). The Pim-1 kinase phosphorylates Ser422 of the forkhead domain of human FOXP3, which attenuates FOXP3 DNA binding activity and down-regulates expression of Treg feature genes. Knockdown of Pim-1 in Tregs enhances suppressive activity (95). These findings demonstrate that phosphorylation of Foxp3 by Pim-1 and Pim-2 negatively regulates Foxp3 transcriptional activity and Treg suppressive function. Kaempferol is a natural flavonoid found in vegetables and fruits, which may reduce PIM1-mediated FOXP3 phosphorylation at S422 and enhance Treg cell suppressive capability. The anti-inflammatory effect of *Kaempferol* may facilitate therapies of certain autoimmune diseases (96).

Phosphorylation of the Ser-418 located in the FOXP3 C-terminal forkhead domain plays a positive role in regulating Treg suppressive function (43). TNF-α induces protein phosphatase 1 (PP1), which can dephosphorylate Ser-418 of FOXP3 and limit Treg cell activity, while increasing pathogenic Th17 and Th1 CD4 T cells within the inflamed synovium. TNF-α antagonist therapy (treatment with TNF-α-specific antibody: Infliximab) increased Treg cell function in patients with rheumatoid arthritis (43). Ser-418 phosphorylation of FOXP3 affected C-terminal cleavage of Foxp3 protein by proprotein convertase (PC) (97) and also modulated Foxp3 DNA affinity by interactions with other proximal modification, such as acetylation (98). FOXP3 can be phosphorylated at the Tyr-342 site by Lymphocyte-specific protein tyrosine kinase (LCK) in the MCF-7 cell line. Tyr-342 phosphorylation of FOXP3 down-regulates expression of *SKP2, VEGF-A*, and *MMP9* genes (99). Taken together, Foxp3 undergoes phosphorylation modifications, and these modifications can either positively or negatively modulate Treg function according to the various disease settings.

### O-GlcNAcylation Modification of Foxp3

Protein O-GlcNAcylation modification occurs at serine and threonine residues, as well as phosphorylation, which is catalyzed by O-GlcNAc transferase (OGT) and O-GlcNAcase (OGA) oppositely. O-GlcNAcylation may counteract ubiquitination to stabilize FOXP3 protein, and loss of O-GlcNAcylation destabilizes FOXP3 protein. Deficiency of OGT in Treg cells leads to lethal autoimmune diseases in mice (100).

### Acetylation of Foxp3 by Histone Acetyltransferases

Acetylation occurs on histone proteins and other cellular proteins (101). Protein acetylation is catalyzed by histone acetyltransferases (HTAs) and histone/protein deacetylases (HDACs) oppositely. HATs can transfer the acetyl moiety of acetyl-coenzyme A to N^α^-amino groups of methionine residue or the N^ε^-amino groups of lysine residues on proteins. N^α^-acetylation occurs as a co-translational process of protein N-terminal methionine cleavage (102), and is a reversible process that modulates protein biological activity in response to internal or external cell stimuli (103).

Based on structural and functional similarity of their catalytic domains, HATs can be grouped into three main families: (1) the Gcn5/PCAF family including Gcn5, PCAF, and related proteins; (2) the p300/CBP family; (3) the MYST family are involved in control of transcription and cell growth and survival (104).

TIP60, p300, and CBP, orchestrate multiple aspects of Treg development, function, and lineage stability (105). TIP60 belongs to the MYST HAT family. The TIP60 histone acetylase complex also plays an important role in DNA repair and apoptosis (106). TIP60 can act as both a transcriptional co-repressor (107, 108) and a co-activator (109). TIP60 is the first identified HAT that acts as an essential subunit of the FOXP3 repression complex. The N-terminal 106–190 aa of FOXP3 associates with TIP60 and HDAC7. The FOXP3–TIP60–HDAC7 complex is required for IL-2 repression by Foxp3 in T cells (110).

p300 acetylates Foxp3 to prevent proteasome-mediated degradation and increase Foxp3 protein levels, as well as Foxp3-mediated transcriptional repression of IL-2 production (111). Mass spectrometry analysis has identified three acetylation sites in murine Foxp3 (K31, K262, and K267), upon co-expression with p300 acetyltransferase in 293T cells (112). A conditional deletion of *Ep300* (which encodes p300) in Treg cells compromises Treg suppressive capability and leads to autoimmunity at around 10 weeks of age. On the other hand, deficiency of p300 impairs Treg cell infiltration into tumor, accompanied with enhanced anti-tumor immunity (113). Administration of the p300 inhibitor C646 or a peptidic p300i (Lys-CoA-Tat) abrogates Treg cell–dependent allograft survival, while increasing the anti-tumor immune responses in animal tumor models (113, 114).

CBP, a p300 paralog, is also important in regulating Treg function in certain inflammatory or lymphopenic conditions. Double-deletion of *CBP* and *p300* in Treg cells leads to fatal autoimmunity by 3–4 weeks of age (115). Likewise, TIP60 and p300 cooperatively regulate FOXP3 activity. p300 interacts with TIP60 and facilitates autoacetylation of TIP60 at K327. This modification increases TIP60 protein stability and promotes FOXP3 acetylation. Reciprocally, TIP60 promotes p300 acetylation and HAT activity as well (116). In contrast to the modest phenotype of *p300*-deficient mice, a deficiency of TIP60 in Tregs results in severe and fatal autoimmune diseases at an early age. These studies indicate that TIP60 plays a more unique and dominant role than p300 (for which there are similar and redundant HAT), in terms of regulation of Treg cell development and function (116). A recent study by Bin Dhuban et al. elegantly shows that some inheritable mutations in the forkhead domain of FOXP3 can specifically disrupt FOXP3-TIP60 association, in turn, compromising human Treg cell development and function. Restoring FOXP3-TIP60 in this setting with allosteric modification of TIP60 also rescued Treg cell function (117, 118).

**Figure 2:**
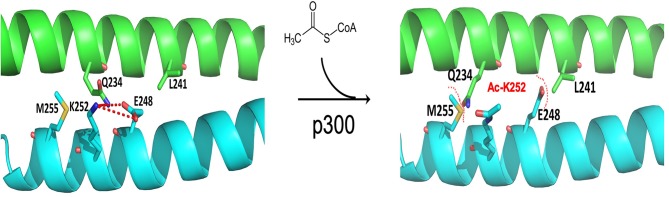
Structure model of acetylation at FOXP3 K252 downregulating suppression by destabilizing homodimerization. FOXP3 forms dynamic homodimer via zinc-finger and leucine-zipper domains, which featured as a two-stranded, anti-parallel a-helical coiled-coil. The inter-subunit hydrogen bond formed between Q234 and E248 stabilizes FOXP3 dimer via electrostatic interactions. The Q234-E248 hydrogen bond depends on the unique conformation of the E248 side chain held by K252 residue **(left)**. Acetylation of K252 by p300 neutralizes the positive charges of K252 side chain and decreases its interaction with E248, and as a consequence, breaks the Q234-E248 inter-subunit hydrogen bond. E248-L241 and Q234-M255 form the steric tension to relax and destabilize the FOXP3 homodimerization **(right)**. (Xiaomin Song, Guoping Deng, and Mark I. Greene).

FOXP3 forms dynamic homo- or hetero- dimer via its zinc-finger and leucine-zipper domain. This dimer structure is characterized as a two-stranded anti-parallel a-helical coiled-coil. The crystal structure indicates that lysine residues, K250 and K252 of FOXP3, are electrostatically involved in the interface network for Foxp3 coiled-coil dimerization. Acetylation of K250 and K252 of FOXP3 by p300 results in dimer relaxation and down-regulates Foxp3 suppressive activity (119). Of note, FOXP3 acetylation and related chromatin DNA binding activity were induced by treatment of TGF-β, through residues other than K250 and K252 (86, 119). These studies suggest a work model that regulates the Treg suppression by integrating the conformation, dimerization and post-translational modification of the FOXP3 protein (Figure 2).

### Deacetylation of Foxp3 by Histone Deacetylation Enzymes

Eighteen human histone deacetylation enzymes (HDACs) have been identified and grouped into four subsets based on their molecular phylogenetic analysis of primary structure, intracellular localization, and homology to yeast counterpart enzymes (120). Mechanistically, the classical (including classes I, II, and IV) HDAC enzymes depend on zinc ions (Zn^2+^), whereas the Sirtuins (class III) HDACs utilize nicotinamide adenine dinucleotide (NAD^+^) as a coenzyme (121).

Treg cells express 11 classical Zn^2+^-dependent HDACs, and several NAD-dependent HDACs belonging to the Sirtuin family. Although targeting HDAC enzymes in mediating multiple aspects of Treg cell function has received extensive attention (122–124), we focus on reviewing the direct effects of HDAC on Foxp3 protein acetylation and related Treg function regulation. Treatment with a pan-HDACi, trichostatin-A (TSA) boosts the production of thymic Foxp3^+^ Treg cells, Treg feature gene expression, Treg suppression and Foxp3 protein acetylation. Combined therapy with TSA and rapamycin (TSA-RPM) can extend cardiac or islet allograft survival in animal models (75). Moreover, HDAC inhibitors, including sodium butyrate, valproate acid, SAHA, MS-275, Bufexamac, and BML210, can increase human Treg suppression (125, 126).

HDAC inhibitor therapy has been examined for its ability to modify Treg function *in vivo* and effects on protection of allograft and prevention of autoimmunity (127). The class III HDAC SIRT1 also decreases Foxp3 acetylation and Treg stability. Pharmacological treatment with either the HDACs pan-inhibitor, TSA, or SIRT specific inhibitor, NAM, increases Foxp3 levels and Treg suppressor capacity in an *in vitro* suppression assay. Treatment with the SIRT activator, resveratrol, decreases numbers of FOXP3^+^ cells in human PBMC and skin samples (111). Deficiency of Sirt1 promotes Foxp3 stability in iTreg cells and restrains the generation of pathogenic T cells. Administration of the Sirt-1 inhibitor Ex-527 attenuates GVHD but preserves the therapeutic effect of graft-vs.-leukemia (128).

HDAC6, HDAC9, and Sirt1 may deacetylate Foxp3, however, these HDACs regulate Foxp3 gene expression by affecting different transcription factors. HDAC6, HDAC9, and Sirt1 have shared and individual mechanisms of action, and the combined loss of HDAC6, HDAC9, and Sirt1 activity may augment Treg function (129–131).

### Ubiquitylation of Foxp3

Protein ubiquitylation modification is catalyzed by a sequential and concerted action of three distinct classes of enzymes: the ubiquitin-activating enzyme, E1, the ubiquitin-conjugating enzyme, E2, and the ubiquitin-ligase, E3 (132). Ubiquitin consists of a 76-amino acid polypeptide that has seven lysine residues (K6, K11, K27, K29, K33, K48, and K63), each of which may participate in the formation of diverse poly-ubiquitin chains. K48-linked polyubiquitin chains represent targeting signals for proteasomal degradation, while K63 polyubiquitylation plays non-proteolytic roles, including DNA damage response, receptor endocytosis and protein trafficking (133).

Ubiquitylation signals regulate multiple aspects of immune cell function and immune response (134). Upon T cell activation, the non-degradative ubiquitylation, including K29, K33, and K63 polyubiquitylation, increases largely in CD4^+^ T cells, which suggests the importance of non-degradative ubiquitylation in T cell signaling (135).

As the master regulator of the Treg cell lineage, Foxp3 protein level and turnover rate determine Treg cell identity and function. Inflammatory stress destabilizes Treg cell functions (136, 137). The Foxp3 protein undergoes polyubiquitylation and proteasome-based degradation especially under certain stressful conditions. HIF-1α promotes expression of Th17 signature genes and Th17 cell development, but attenuates Treg development by enhancing Foxp3 ubiquitylation and proteasomal degradation (138). Stimulation by proinflammatory cytokines and lipopolysaccharides (LPS) promotes Foxp3 K48-linked polyubiquitination by the E3 ubiquitin ligase, Stub1, in an Hsp70-dependent manner. The absence of endogenous activity of Stub1 or Hsp70 prevents Foxp3 from proteasomal degradation (139).

Upon TCR stimulation, E3 ubiquitin ligase, Cbl-b, together with Stub1, targets Foxp3 for ubiquitylation and degradation; and a deficiency of Cbl-b can partially rescue defective development of thymic Treg cells in *Cd28*^−/−^ mice (140). Cimetidine (CIM) can promote immune responses, suppress Treg cell function via activation of PI3K-AKT-mTOR signaling and increase of Stub1-mediated degradation of Foxp3, respectively (141). As mentioned above, Foxp3 phosphorylation by NLK prevents its association with the STUB1 and increases Foxp3 protein stabilization (87). These studies highlight the critical role of Stub1 ubiquitin E3 ligase in mediating Foxp3 protein level and Treg suppressive function.

Besides K48-linked ubiquitylation, Foxp3 also undergoes non-proteolytic ubiquitylation. Tumor necrosis factor (TNF) receptor-associated factor 6 (TRAF6) mediates K63-linked polyubiquitylation (142). TRAF6 plays an essential role in maintaining Treg cell function to inhibit Th2 type autoimmunity (143). A recent study found that deficiency of TRAF6 in Treg cells compromises Treg suppression *in vivo*. Mechanistically, TRAF6 interacts with FOXP3 and mediates K63 polyubiquitination at lysine 262 residue, which ensures proper nuclear localization of FOXP3 and facilitates FOXP3 transcriptional activity in Tregs. Deficiency of TRAF6 in Tregs enhances anti-tumor immunity (144). The E3 ubiquitin ligase ring finger protein 31 (RNF31) catalyzes FOXP3 atypical ubiquitylation, and promotes FOXP3 protein stability and Treg suppression. RNF31 expression has been correlated with intratumoral Treg cell activities in gastric cancer, indicating a potential role of RNF31 in tumor immunity (145).

### Deubiquitylation of Foxp3

Protein ubiquitylation can be reversed by the deubiquitinases (DUBs, also known as deubiquitinating enzymes) (146). The deubiquitinases consist of five families: ubiquitin C-terminal hydrolases (UCHs), ubiquitin-specific proteases (USPs), ovarian tumor proteases (OTUs), Josephins and JAB1/ MPN/MOV34 metalloenzymes (JAMMs). The UCH, USP, OTU, and Josephin families are Cys proteases, whereas the JAMM/MPN+ family members are zinc metalloproteases (147).

It has been well-documented that deubiquitinases are involved in T cell development, activation, differentiation and tolerance (148). The K63-specific deubiquitinase Cylindromatosis (CYLD) negatively regulates CARMA1, which is required for NF-kB activation and IL-2 receptor signaling. A deficiency of CYLD causes constitutive NF-kB activation and enhanced TGF-b signaling, which increases the frequency of Treg cells in peripheral lymphoid organs and promotes Treg cell differentiation *in vitro* (149, 150). Similarly, Treg cell number increases in a non-functional CYLD splice variant CYLD^ex7/8^, however, the suppressive capacity is impaired and correlated with decreased expression of CD25 and CTLA4 (151).

USP7 is upregulated in Treg cells, and the ectopic expression of USP7 decreases Foxp3 polyubiquitylation and increases Foxp3 expression. Treg cells pretreated with deubiquitinase inhibitor enhance their modifying functions in adoptive transfer induced colitis (152). In addition, USP7 promotes Treg suppression by enhancing the multimerization of Tip60 and Foxp3 (153). USP21 associates with GATA3 and Foxp3 transcriptional factors. Mice depleted of Usp21 in Treg cells develop Th1-type inflammation (154, 155). USP4 stabilizes the IRF8 protein via a K48-linked deubiquitinase, which promotes the suppressive function of Treg cells (156).

### Methylation of Foxp3

Arginine methylations are mainly catalyzed by the Protein Arginine Methyltranasferase (PRMT) family. PRMT family can be divided into 3 subfamilies: the Type 1 PRMTs include PRMT1, 2, 3, 4 (also known as CARM1), and 6, which asymmetrically methylate target arginine residues; the Type 2 PRMTs include PRMT5 and 9, which symmetrically methylate target arginine residues; the Type 3 PRMT7 mono-methylates target arginine residues (157, 158).

T cell activation induces protein methylation modification (159). Employing isomethionine methyl-SILAC (iMethyl)-SILAC and mass spectrometry approaches, various transcription factors, including Foxp3, that influence T cell differentiation and lineage specificities, have been identified as being methylated (160). FOXP3 can be di-methylated at arginine(R) 51 position, although whether this methylation is asymmetry or symmetry, and which PRMT is responsible for this modification both remain unclear (129). More recent mass spectrometric analysis reveals that human FOXP3 contains several possible di-methylation sites at R27, R51, and R147 residues (161). PRMT1 and PRMT5 are reported to be involved in FOXP3 methylation (162). PRMT5 preferentially binds to FOXP3, and a conditional deletion of PRMT5 gene in Tregs leads to deadly scurfy-like autoimmune diseases. Lymph node Tregs from those animals showed significantly less suppressive function, indicating that PRMT5 is essential for Treg function. Site-mutation of R51K dramatically decreases symmetric arginine signals, as detected by symmetric arginine specific antibody sym10, suggesting that PRMT5 di-methylates FOXP3 at R51. Of note, the arginine methylation at the N-terminal position of FOXP3 regulates Treg function via a Foxp3-DNA-binding-activity independent manner. Pharmacological ablation of PRMT5 activity by DS-437 can enhance the anti-tumor efficacy of anti-erbB2/neu monoclonal antibody target therapy by reducing human Treg functions (161).

Targeting protein methylation modification provides novel insights into clinical therapeutic designing for certain diseases, including autoimmune diseases and cancer. PRMT5 forms a complex with MEP50, an essential cofactor, which then causes higher enzymatic activity and allows the binding to target proteins. PRMT5 uses S-adenosyl methionine (SAM) for transferring a methyl group to the target substrate. It is also known that PRMT5 forms a large complex with different cofactors, for changing its target proteins (158). PRMT5 is highly expressed in a variety of cancers, and several inhibitors have been developed that target PRMT5 for cancer therapy (157). PRMT5 forms a large complex and has several different cofactors; the effect of PRMT5 inhibitors may be dependent on the screening strategy, such as substrate- or SAM- competitive. SAM-competitive inhibitor, DS-437, has more efficient inhibition of FOXP3 methylation than the substrate-competitive inhibitor, EPZ015666 (161). Thus, for targeting FOXP3 methylation in Treg cells, it may rationalize to use SAM-competitive inhibitors. Likewise, Foxp3 associates with PRMT1 and is asymmetrically di-methylated at R48 and R51 positions. FOXP3 methylation and its function is compromised by administration of PRMT1/6 inhibitor MS023 (162). Based on the current findings, more research is needed to clarify the biological significance of the asymmetric methylation on FOXP3 in modulating Treg suppression.

## In Summary

Both lymphoid-organ Treg cells and tissue-resident Treg cells require the expression of the Foxp3 transcription factor. Foxp3 protein functions in transcriptional molecule complexes. Ensemble formation is regulated by various interactions and post-translational modifications (PTMs), including phosphorylation, O-GlcNAcylation, acetylation, ubiquitination, and methylation. Modifications influence each other to orchestrate Foxp3 activity and Treg suppression. However, the currently available, published results are still not sufficient to fully reveal roles of Foxp3 PTMs in modulating Treg suppression, and some caveats should be considered when interpreting the data. For example, to what extent does the ectopic expression of protein in the transfected cell line represent the real situation in Treg cells? Are there potential substrates other than Foxp3 responsible for the phenotypes observed in those enzyme-deficient mice? What are the possible cross-talks among different Foxp3 PTMs under certain tissue- microenvironment? What is the clinical relevance and significance of Foxp3 PTMs? Moreover, are there other types of post-translational modifications occurring on Foxp3 that have not been identified yet, such as lysine crotonylation or succinylation?

The understanding of Foxp3 protein PTMs in modulating Treg suppression may facilitate the design of rational therapies for immune disorders developed in IPEX patients. Based on the high-resolution crystal structure of Foxp3, combination therapy by targeting Foxp3 post-translational modifications with other therapies may enhance therapeutic approaches in a variety of diseases. Moreover, a full understanding of the contribution of other regulatory cells, such as Suppressor T cells, may lead to additional therapeutic alternatives (163–165).

## Author Contributions

All authors listed have made a substantial, direct and intellectual contribution to the work, and approved it for publication.

### Conflict of Interest

SF is employed by company Seishin Medical Group, Takara Clinic. The remaining authors declare that the research was conducted in the absence of any commercial or financial relationships that could be construed as a potential conflict of interest. The reviewer ZZ declared a shared affiliation, with no collaboration, with the author XS to the handling editor at the time of review.
